# Light transmission aggregometry using pre-coated microtiter plates and a Victor X5 plate reader

**DOI:** 10.1371/journal.pone.0185675

**Published:** 2017-10-12

**Authors:** Pernille Just Vinholt, Mads Nybo, Camilla Brødsgaard Nielsen, Anne-Mette Hvas

**Affiliations:** 1 Department of Clinical Biochemistry and Pharmacology, Odense University Hospital, Odense, Denmark; 2 Department of Clinical Biochemistry, Aarhus University Hospital, Aarhus, Denmark; Institut d'Investigacions Biomediques de Barcelona, SPAIN

## Abstract

**Background:**

Light transmission aggregometry (LTA) can be performed with microtiter plates (96-well LTA). When conducting LTA, an agonist is added to platelet-rich plasma and the sample is shaken for minutes after which absorbance readings are done. Platelet aggregation is detected as decrease in absorbance. However, the classical method is cumbersome and therefore microtiter plates can be used for concomitant testing of multiple samples. Furthermore, it would be convenient to prepare the plate in advance of platelet aggregation testing. Aim: The aim of the present study was to establish a simplified 96-well LTA protocol, where plates were pre-coated with agonists and stored at -80 C until use.

**Results:**

We developed and validated a protocol for 96-well LTA using a Victor X5 plate reader and pre-coated microtiter plates. The minimum requirement of platelet-rich plasma was 45 μL per sample and the sample platelet count should not be below 100 x109/L. Optimal absorbance reading was 595 nm wavelengths. Platelet aggregation results were higher at 37°C than at room temperature. Platelet adherence to wells after stimulation was observed; it was not avoided by pre-coating of the wells with gelatin. A range of up to 7 concentrations for each agonist (collagen, arachidonic acid, adenosine diphosphate, thrombin receptor-activating peptide and protease-activated receptor-4) was tested concomitantly. A transient rise in platelet aggregation was observed after 2 minutes of shaking in some samples with low agonist concentration, and platelet aggregation was optimal after 10 minutes of shaking for samples with high agonist concentration. Plates could be stored at -80°C for 15 days without significant change in the platelet aggregation results.

**Conclusion:**

The 96-well LTA is suitable for platelet aggregation testing and a range of agonist concentrations can be concomitantly tested.

## Introduction

Platelets are essential in primary haemostasis and are therefore also directly involved in the pathophysiology of arterial thrombosis and bleeding [1, 2]. Light transmission aggregometry (LTA) is the gold standard for evaluating platelet function and is based on the principle that light transmission increases with platelet aggregation [3]. Classical LTA is performed on aggregometers with up to eight channels, making it impractical to test multiple samples or conditions. Further, multiple testing is often not feasible within the recommended time span for platelet function testing (up to 4 hours from sample collection), and large sample volumes are required [3]. Microtiter plates are widely used for immunological testing and for cell culturing, and it has been proposed that LTA can be scaled down and performed in 96-well microtiter plates [4–12]. The advantage of 96-well aggregometry is that large numbers of samples can be tested simultaneously as the required blood volume per sample is significantly smaller. However, it may be impractical to prepare the plates immediately before platelet function testing. Recently, it was shown that plates could be pre-coated, agonists lyophilized, and plates kept at room temperature before use [10–12]. This technique is, however, not available at all laboratories.

The aim of the present study was to establish and evaluate an approach, where 96-well aggregometry was conducted using pre-coated plates stored at -80°C until required. The study will additionally provide documentation for aspects that not are not covered in the current literature, including a) whether there exists a linear relationship between platelet count and optical densities in the microtiter wells, b) whether protease activated receptor (PAR)-4 is suitable as agonist in 96-well LTA, c) coefficient of variation (CV%) for all agonists for concentrations used when determining dose-response curves, d) report on a detection limit for the assay in terms of the lower limit for the platelet count, and e) testing if platelet adherence occurs during platelet aggregation, and whether it then can be prevented with plates pre-coated with gelatin.

## Material and methods

### Protocol for 96-well aggregometry

Five microlitre (μL) of phosphate-buffered saline (PBS) with or without agonist was added to individual wells of half-area 96-well microtiter plates (Greiner Bio-One, Stonehouse, Gloucestershire, UK). The plates were sealed and stored at -80°C. Platelet agonists were arachidonic acid (AA; 0.03–1 mM), thrombin receptor-activating peptide (TRAP, SFLLRN, 0.3–32 μM) and collagen Type 1 (0.01–30 μg/mL), adenosine diphosphate (ADP, 0.12–40 μM), and PAR-4 agonist (AYPGKF- NH_2;_ 6.25–200 μM). ADP was from Sigma Aldrich (St. Louis, Missouri, USA) and PAR-4 was from Bachem (Bubendorph, Switzerland), while other agonists were from Roche Diagnostics (Mannheim, Germany).

All samples originated from healthy individuals. For platelet function testing, blood was drawn by venipuncture from a peripheral vein. For the platelet aggregation assay we used 3.2% (109 mM) trisodium citrate anticoagulated blood in BD vacutainer tubes (Becton Dickinson, New Jersey, USA). EDTA (1.8 mg/mL)-anticoagulated blood was used in few experiments and stated explicitly when used.

Blood was centrifuged at 200 *g* for 10 minutes and platelet-rich plasma (PRP) was collected. The remaining sample was re-centrifuged at 10.000 *g* for 10 minutes and platelet-poor plasma (PPP) was collected. Upon testing, 45 μL of PRP was added to wells with agonists. As reference, 45 μL of PRP or PPP was added to wells with PBS. Plates were shaken (900 rpm, orbital shaking) for 10 minutes at 37°C and thereafter, absorbance was measured using Victor X5 (Perkin Elmer, Turku, Finland) at 595 nm and results reported as optical density (OD). Platelet aggregation was calculated from OD in wells with agonists in reference to PRP (set to 0% aggregation) and PPP (set to 100% aggregation).

$$Platelet\;aggregation=\frac{\left(OD\;PRP-OD\;sample\right)}{\left(OD\;PRP-OD\;PPP\right)}*100\%$$

Regardless the sample volume, agonists comprised 10% of sample volume in all tests. If not otherwise stated, experiments were conducted with the stated protocol using a total sample volume of 50 μL per experiment and freshly prepared plates. The optimal discrimination of PRP from PPP was found by determining the ratio between OD in PRP and OD in PPP. We measured absorbance at different wavelengths and sample volumes (30–150 μL) to evaluate this ratio and thus find appropriate settings, n = 9. The relationship between platelet count and OD in unstimulated samples was determined by measuring preparations of PRP diluted with PPP. Different sample volumes were compared (25–180 μL), performed in duplicate on a pool from three healthy individuals. Detection limit was evaluated by comparing platelet aggregation in samples with varying platelet counts and determining the CV% of intra-serial platelet aggregation measurements of samples with varying platelet counts. For this purpose, samples with platelet counts ranging 50–300 x10^9^/L were constructed, n = 5. This was done by isolation of platelets as previous described [13]. Isolated platelets were then resuspended in PPP and samples were stimulated with 6.4 μM ADP.

The following experiments were conducted using a high and a low concentration of each agonist (arachidonic acid 0.125 mM and 1 mM; ADP 1.1 μM, 6.4 μM or 12.8 μM; TRAP 1.1 μM and 10 μM; collagen 1.1 μg/L, 6.4 μg/L or 12.8 μg/L, and PAR4 50 μM and 100 μM). We evaluated the effect on platelet aggregation of temperature at 22°C versus 37°C, n = 9. The degree of platelet adhesion was evaluated in plates after sample processing according to the protocol; after platelet aggregation, samples were removed and wells were washed once with PBS before absorbance readings; ODs in wells emptied of PRP after platelet aggregation were compared with wells that previously contained unstimulated PRP or PPP. The effect of pre-coating with gelatin in PBS, 0.75% weight per volume (W/V) was determined, n = 9 per setting. Moreover, the effect of shaking on OD was evaluated in unstimulated samples, n = 9 per setting. We compared the effect of shaking on ODs in PRP from citrate versus EDTA-anticoagulated blood.

We evaluated the effect on platelet aggregation of shaking for up to 20 minutes, n = 3. The response curve from 10 individuals was obtained. Lyophilized aspirin (Selleckchem, Houston, Tx, USA) was suspended in DMSO according to the manufacturer’s instruction and further diluted in PBS. Aspirin-treated samples were used as a control for the assay sensitivity, n = 3. The samples were pre-incubated for 30 minutes at 37°C with aspirin 40 μM or vehicle (DMSO in PBS). Intra-serial CV% was determined using 20 samples (10 samples from two individuals with normal platelet count). The effect of storage was tested using plates stored for varying time before processing.

### Ethics

All participants gave written informed consent. The study was approved by the Regional Scientific Ethical Committees of Southern Denmark and the Danish Data Protection Agency, and was conducted in accordance with the guidelines of the Declaration of Helsinki.

### Statistics

Categorical variables are reported as percentages. Continuous variables are given as mean and standard deviation or median and interquartile ranges (IQR). Group-comparisons were conducted with t-test or Mann-Whitney U test for non-normally distributed data, as appropriate. Statistical analyses were performed using GraphPad Prism 5.0 (GraphPad Software, La Jolla, CA, USA). P-values were two-sided.

## Results

**Fig 1:**
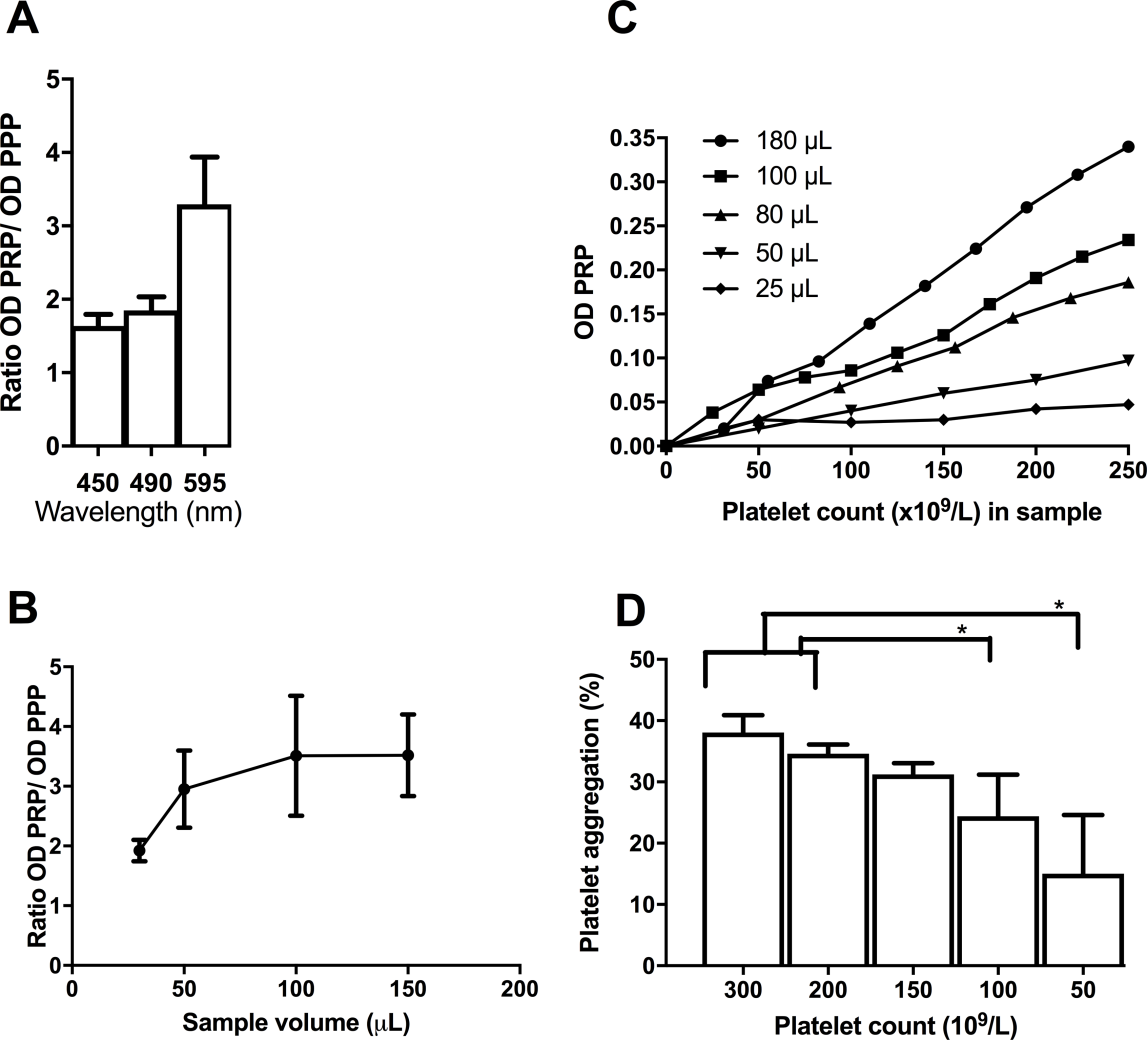
The effect of wavelength and sample volume on absorbance measurements. Experiments were conducted at 37°C. Results in figure A-C is from unstimulated samples and measurements are performed without shaking. a) Shows the ratio between optical density in platelet-rich plasma (PRP) and optical density in platelet-poor plasma (PPP) when comparing wavelengths for absorbance measurements. Results are mean and SD from triplicate samples from three persons). b) Shows the ratio between optical density in PRP and optical density in PPP dependent on sample volume. Results are mean and SD from triplicate samples from three persons. c) Shows the effect of platelet count in PRP on absorbance measurements at different sample volumes. Results are mean of duplicates on a pool from three persons. d) Shows platelet aggregation results from samples with platelet counts ranging 50–300 x10^9^/L constructed from isolated platelets suspended in autologous PPP and stimulated with 6.4 μM adenosine disphosphate, n = 5. * indicates p<0.05. Results are mean and SD. Group comparisons were done with the t-test.

The 595 nm wavelength gave the best discrepancy between PRP and PPP in terms of the highest ratio between OD (Fig 1A); the ratio was highest for sample volumes above 30 μL (Fig 1B). There was a linear relationship between OD and platelet count in PRP at all sample volumes. Higher ODs were observed at increasing sample volumes when comparing samples with equal platelet counts (Fig 1C). When using 50 μL samples we found a significant decrease in platelet aggregation results when PRP contained ≤100 x10^9^/L platelets as compared to platelet count >150 x10^9^/L in samples constructed from the same blood samples, p = 0.001 (Fig 1D). Further, the CV% was 128% at 50 x10^9^/L and 56% at 100 x10^9^/L versus 9–15% at platelet count ≥ 200 x10^9^/L.

Platelet aggregation was higher at 37°C compared with 22°C, 86% ±4 versus 81% ±4, p = 0.02 for ADP 12.8 μM; 80% ±3 versus 72% ±4, p = 0.001 for collagen 12.8 mg/mL, and 92% ±3 versus 84 ±3, p<0.001 for PAR-4 100 μM, respectively. No difference was observed for arachidonic acid and TRAP-induced platelet aggregation.

**Fig 2:**
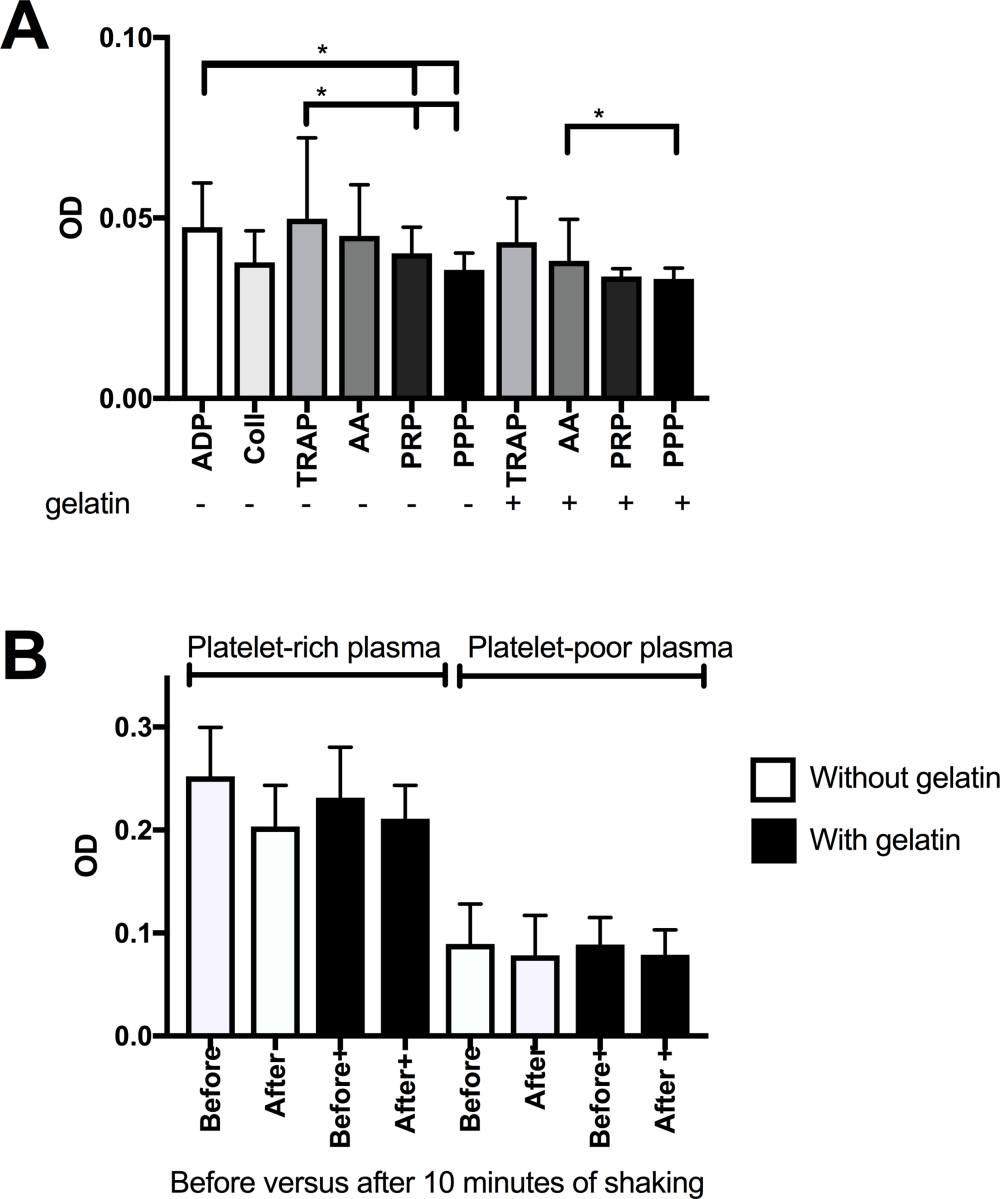
The effect of platelet adhesion and plate shaking on absorbance readings. Experiments were conducted at 37°C and when relevant 10 minutes of shaking was used. a) Platelet adhesion in wells is detected by absorbance readings in wells emptied after platelet aggregation. It is compared to absorbance readings in wells previously containing unstimulated platelet-rich plasma (PRP) or platelet-poor plasma (PPP). Platelet adhesion in wells pre-coated with gelatin (+) is compared to wells without gelatin (-), results are mean and SD, n = 9 (triplicate samples from three persons). * indicates p<0.05. Group comparisons were done with the t-test. ADP, adenosine diphosphate; OD, optical density; TRAP, thrombin receptor-activating peptide; AA, arachidonic acid. b) The effect of shaking on absorbance readings in PRP. Measurements before and after 10 minutes of shaking is compared. Wells pre-coated with gelatin and without gelatin is compared, n = 9. Group comparisons were done with the t-test, none had p<0.05.

We examined whether ODs were influenced by platelet adherence to wells. When wells were washed after ended platelet aggregation, there was some platelet adherence as mean OD was higher in emptied wells that previously contained agonist-stimulated PRP compared to emptied wells that previously contained unstimulated PRP or PPP (Fig 2A). The difference was small, but significant for ADP, TRAP and collagen-stimulated PRP in wells without gelatin and for TRAP-stimulated PRP in wells with gelatin. There was no difference in ODs in wells with versus without pre-coating with gelatin compared to wells that previously contained stimulated PRP or PPP. The OD in wells that previously contained unstimulated PRP equaled wells emptied of PPP, OD 0.03 ±0.01, which means that unstimulated platelets did not adhere to the wells. In all wells that had contained unstimulated PRP, ODs were lower than OD of a (full) well with PPP sample from the same individuals (OD 0.07 ±0.001), p = 0.002.

Shaking for 10 minutes caused an insignificant decrease in OD of unstimulated PRP in plates (Fig 2B). When comparing OD in unstimulated PRP in wells with versus without gelatin, there was no difference in the OD. When using PRP obtained from EDTA-anticoagulated blood, there was no change in ODs after 10 minutes of shaking, ODs were 0.16 ±0.005 versus 0.15 ±0.002, p = 0.2, before and after shaking, respectively.

**Fig 3:**
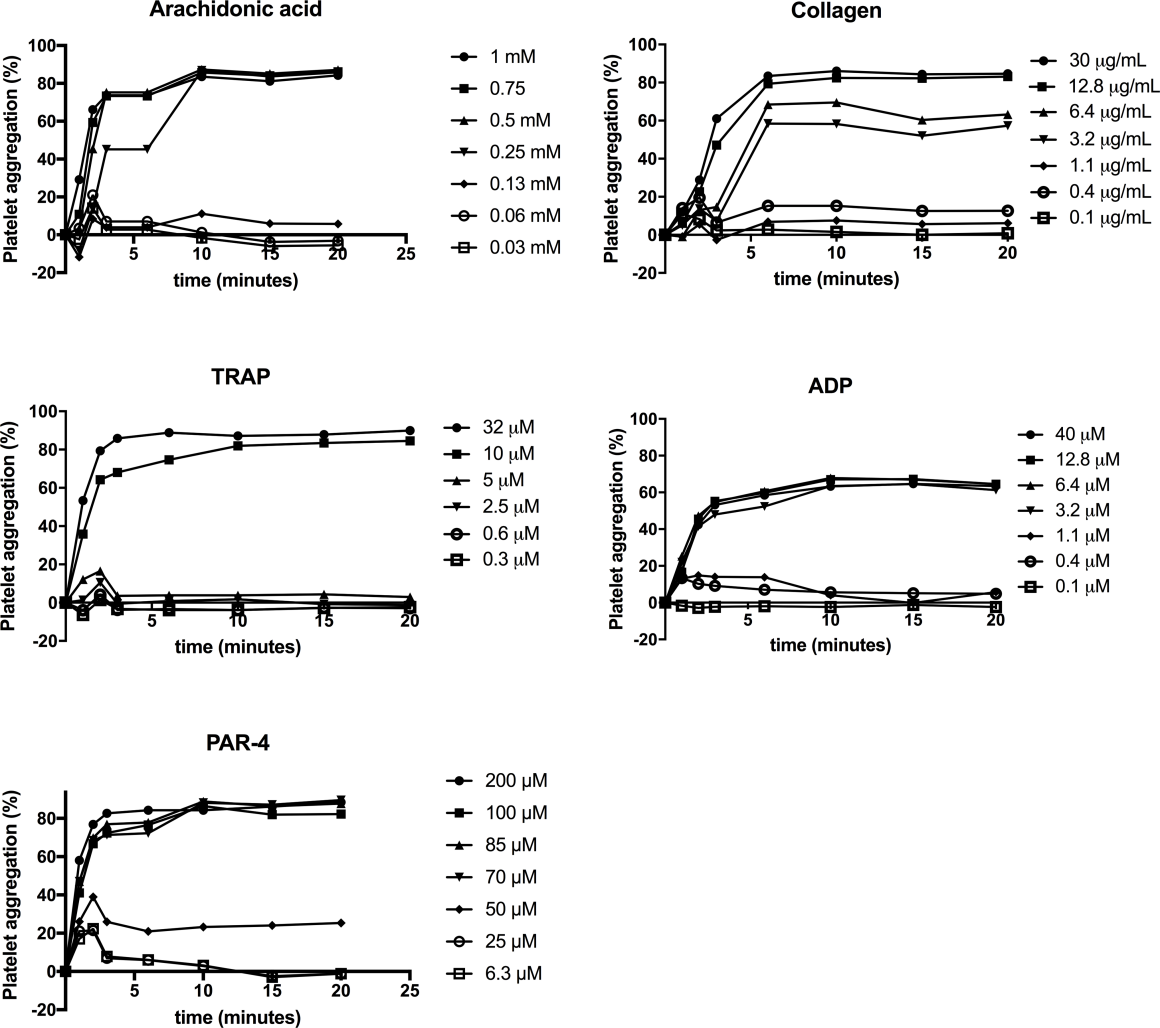
Effect of time of shaking on platelet aggregation results. The effect of time of shaking on platelet aggregation results using different agonists. Except for time settings, the experiments were conducted with the final protocol at 37°C. Each graph represents mean platelet aggregation for one agonist. The curve within a graph represents platelet aggregation as a function of shaking time at different agonist concentrations, n = 3 persons per test condition. Same persons for all agonists. ADP, adenosine diphosphate; TRAP, thrombin receptor-activating peptide; AA, arachidonic acid; PAR-4, protease-activated receptor-4.

**Fig 4:**
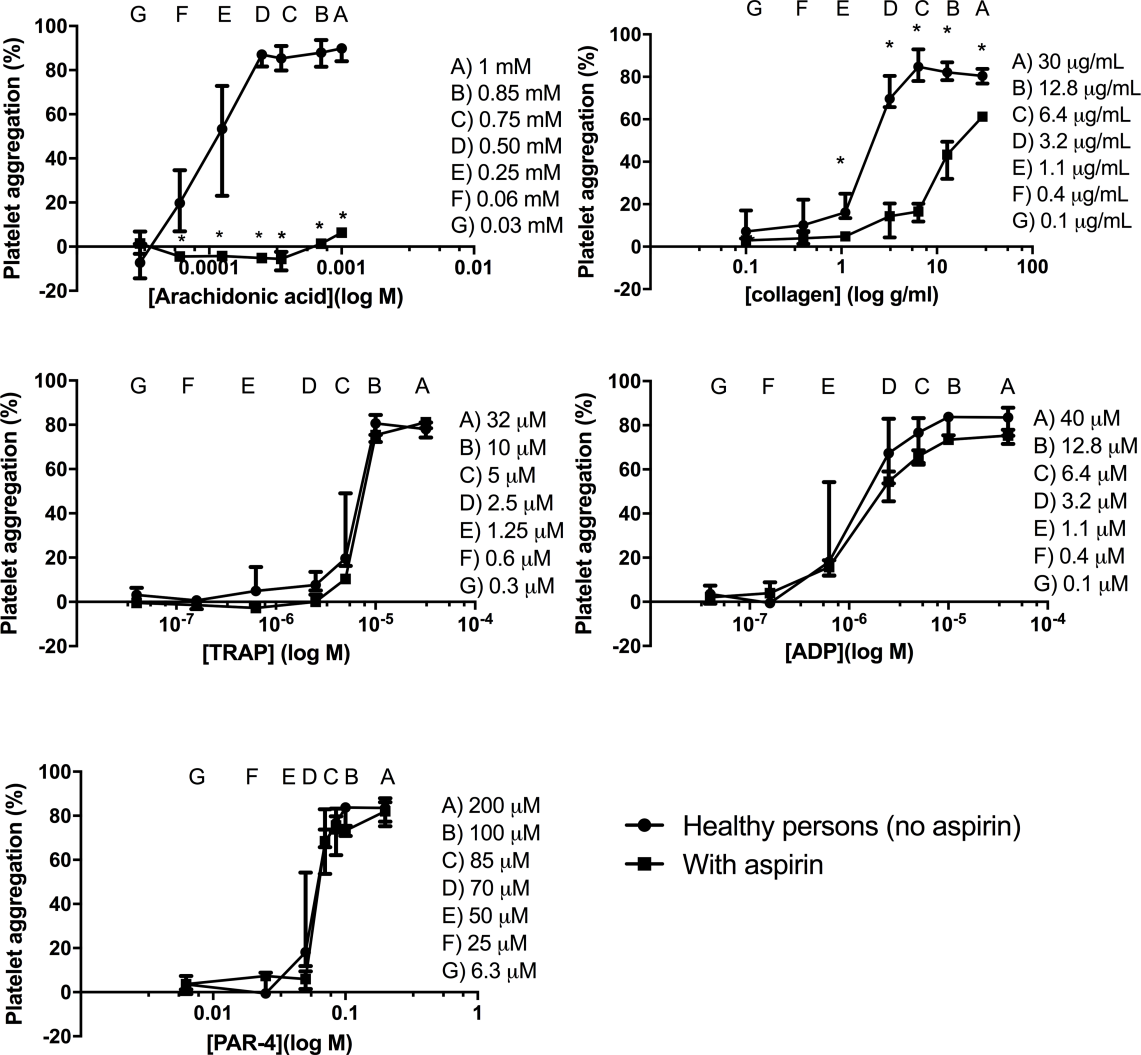
Platelet aggregation in healthy individuals and the effect of aspirin. Platelet aggregation was measured after 10 minutes according to the final protocol at 37°C. Platelet aggregation was tested in 10 healthy individuals, same individuals for all agonists. Median platelet aggregation at a given agonist concentration (circles) and interquartile ranges (whiskers) are shown. Samples from three individuals were pre-incubated with aspirin 40 μM. The graph shows median platelet aggregation (squares) and ranges for this group. * indicates p<0.05. Group comparisons were done with the Mann-Whitney U test. ADP, adenosine diphosphate; TRAP, thrombin receptor-activating peptide; PAR-4, protease-activated receptor-4.

**Fig 5:**
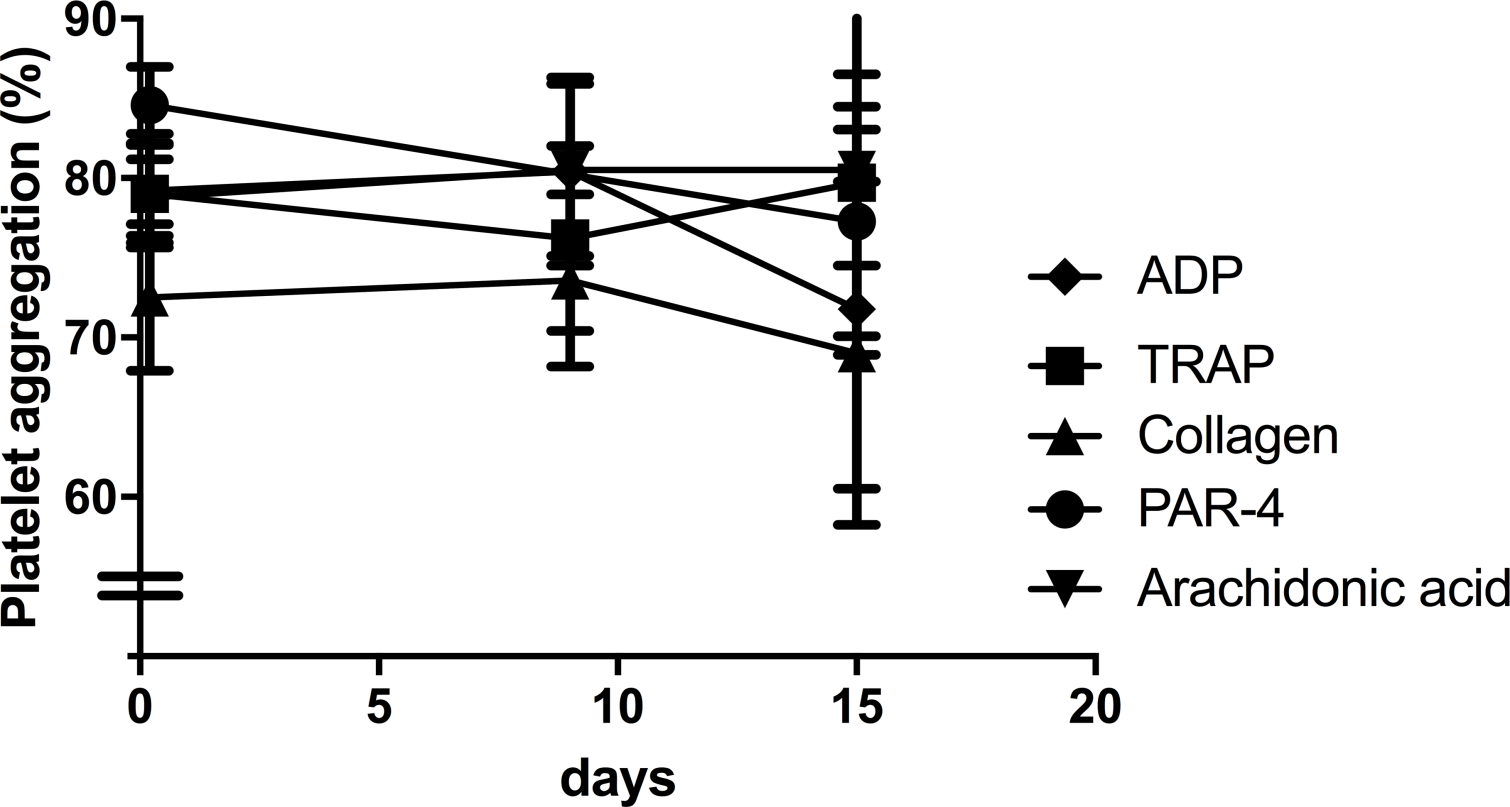
Platelet aggregation in microtiter plates stored at -80°C. The platelet aggregation was evaluated in microtiter plates pre-coated with agonist or buffer, sealed and stored for up to 15 days at -80°C, n = 4 samples. Platelet aggregation was conducted following the final protocol with 10 minutes of shaking at 37°C. ADP, adenosine diphosphate; TRAP, thrombin receptor-activating peptide; AA, arachidonic acid, PAR-4, protease-activated receptor-4.

For several of the agonists in high concentrations, maximal platelet aggregation was reached after 10 minutes of shaking (Fig 3), while for low agonist concentrations a transient rise was observed in some samples resulting in a biphasic response with a peak in aggregation after 1–2 minutes. Platelet aggregation results obtained from healthy individuals are given in Fig 4 and show a clear relationship between agonist concentration and platelet aggregation in healthy individuals. In samples pre-incubated with aspirin, we found that aspirin inhibited arachidonic acid and to lesser extent than collagen-induced platelet aggregation. There was no effect of vehicle on the platelet aggregation as compared to samples from healthy donors (without vehicle). The difference in platelet aggregation between samples with aspirin compared with response of healthy persons was dependent on the agonist concentration. The CV% for intra-serial measurements was low at high agonist concentrations but high at low agonist concentrations (Table 1). Plates were stored up to 15 days without significant decrease in platelet aggregation (Fig 5).

**Table 1:** Coefficient of variation (intra-serial CV%).

Agonist	CV%
TRAP 10 μM	4%
TRAP 2.5 μM	99%
ADP 12.8 μM	1%
ADP 1.1 μM	79%
AA 1 mM	5%
AA 0.25 mM	37%
Collagen 12.0 mg/mL	7%
Collagen 1.1 mg/mL	31%
PAR-4 100 μM	3%
PAR-4 50 μM	17%

Coefficient of variation obtained on samples from two healthy persons with 10 measurements from each person. ADP, adenosine diphosphate; TRAP, thrombin receptor-activating peptide; AA, arachidonic acid, PAR-4, protease-activated receptor-4

## Discussion

We here present a feasible protocol for platelet aggregation test using microtiter plates and a Victor X5 plate reader with use of only 45 μL of PRP per sample into wells pre-coated with agonists and stored at -80°C.

The use of microtiter plates for LTA has previously been evaluated and used in experimental studies [4–12]. However, these studies have not all focused on optimization of the concept and did not provide all details regarding the performance of the assay. In the present study, we did a thorough testing of the concept and initially, we showed that a linear relationship exists between ODs and platelet count in unstimulated samples; a relationship which we did not find any documentation for in prior studies. This association is, however, a prerequisite for using change in ODs as measure of platelet aggregate formation. Also, we found that absorbance reading at a wavelength of 595 nm and with a sample volume of 50 μL was suitable for discriminating PRP from PPP. One prior study did in accordance suggest testing at 563 nm when using plasma as matrix and 405 nm was suitable for washed platelets (5). Even though higher volumes gave better discrepancy between PRP and PPP, we chose to validate the assay using 50 μL per well because the low volume is advantageous for concomitant testing of multiple samples. The results obtained regarding the effect of volume is in accordance with a previous study [11].

Next, we evaluated the test conditions. We found that platelet aggregation was higher at 37°C except for arachidonic acid and TRAP-induced platelet aggregation, which were unaffected by temperature. Prior results are conflicting as arachidonic acid-induced platelet aggregation using 96-LTA was found temperature-dependent [7] and TRAP-induced platelet aggregation was not (5). Nevertheless, we suggest that 96-well aggregometry is conducted at 37°C, which also best reflects normal physiology and is in accordance with what apply for classical LTA [3, 14]. When evaluating the detection limit, we found a significant decrease in platelet aggregation results when PRP contained ≤100 x10^9^/L platelets. Moreover, CV% increased to an unacceptable level. Thus, the present study puts forward that 96-well LTA should not be used with samples with low platelet count. This is in agreement with guidelines for classical LTA [14].

We found that the Victor X5 plate reader is suitable for platelet aggregation testing because it can be programmed to 37°C and to shake the plate as required in order to cause aggregation of stimulated platelets. The plate reader can be programmed to shake and automatically proceed to absorbance readings. While this is definitely easy, it is possible alternatively to shake the plates using a conventional thermo-shaker [11]. Any change in the protocol would possibly affect the aggregation results and it is thus important to include healthy individuals for reference regardless the protocol.

The classical LTA is an approach that specifically evaluates the platelet aggregation which occurs as a result of platelet activation after addition of specific agonists. In classical LTA, it is implicit that platelet adhesion do not impact the results because the assay typically uses glass cuvettes comprising a less adhesive surface than plastic. The current study provides information regarding platelet adhesion in the 96-well LTA approach, where wells are made of plastic. Some have used pre-coating with gelatin before aggregation testing [10], and we tested whether it reduces platelet pre-activation and spontaneous platelet adhesion. We found that during testing some degree of platelet adherence to the well’s wall occurs since stimulated platelets adhere to the wells but unstimulated platelets do not. Adherence does cause a slight increase in ODs which could reduce the calculated platelet aggregation during testing. We put forward that platelet adherence is not avoided by pre-coating with gelatin as the results with and without gelatin were similar and gelatin can therefore be omitted. How to overcome platelet adhesion therefore remains unsolved.

Shaking of the plate without addition of agonists gave a small insignificant decline in ODs, which might partly be due to the viscous PRP covering the side of the well’s wall thereby decreasing the measurements of ODs through the (reduced) liquid column at the middle of the well. A reduction in ODs was not observed with use of EDTA-anticoagulated PRP and might therefore also to some extent be due to spontaneous platelet aggregation. The change in ODs corresponded to about 5% absolutte difference in platelet aggregation response when calculating platelet aggregation based on ODs of PRP obtained prior to and after shaking.

An advantage of 96-well LTA is that multiple samples can be tested simultaneously and thereby multiple agonist concentrations can be used concomitantly as shown in the present study. We also provide documentation that PAR-4 is a suitable agonist in this approach. Maximal platelet aggregation is dependent on agonist concentrations, and transient increase in platelet aggregation can be observed after only few minutes of shaking for agonists in low concentration. This was confirmed by our findings and is in agreement with studies using classical LTA.

Traditionally, high agonist concentrations have been used for bleeding disorder diagnostics with the aim of finding patients with platelet aggregation defects. However, when appliying too high an agonist concentration, a mild defect may remain undetected because the platelets may aggregate properly at very high stimulus [12]. Accordingly, the relative platelet inhibitory effect of aspirin was registered to be lower at the highest concentration of collagen in our study. This is observed as a smaller difference in platelet aggregation response between aspirin-treated and untreated samples at the highest collagen concentration. Thus, the registered platelet inhibitory effects could be counteracted by a high agonist concentration. Based on the present study, we propose that platelet aggregation should be evaluated after 2 minutes and again after 10 minutes of shaking to address both transient and maximal aggregation. However, we also put forward that the CV% is very high at low agonist concentrations, which limits the use of low agonist concentrations on an individual level.

The herein proposed protocol requires only a -80°C freezer and a Victor X5 plate reader (or a thermo shaker and an absorbance reader), which makes the approach feasible for most laboratories. We showed that plates can be stored up to 15 days and therefore can be prepared in advance of testing. The presented microtiter plate approach has several potential applications in both experimental and clinical studies as comparison of multiple conditions and several agonists can be concomitantly tested and thus the relative importance of several factors can be explored.

In some conditions, e.g. in persons with atherothrombotic disease, persons might have an abnormally high platelet aggregation response to agonists. This can only be studied at low agonist concentrations, where the used agonist does not normally result in full aggregation in healthy individuals. Such studies were lacking, but became possible with the microtiter approach since it can be used to study the full dynamic range of agonist concentrations concomitantly.

In conlusion, we show that 96-well LTA can be performed with pre-coated frozen plates using Victor X5 plate reader. We optimized the test and documented the settings including the relationship between OD, platelet count and the dependency on temperature; we provided dose-reponse curves from healthy individuals for a panel of agonists including PAR-4 and provided information regarding the performance. Finally, we found that platelet adhesion does occur, but can not be prevented with pre-coating with gelatin.

## Supporting information

S1 File Data for experiments. [Data_LTA_05022017.xlsx].
(XLSX)

## References

[1] de Groot PG, Urbanus RT, Roest M. Platelet interaction with the vessel wall. Handbook of experimental pharmacology. 2012;(210):87–110. doi: 10.1007/978-3-642-29423-5_4 . 22918728 10.1007/978-3-642-29423-5_4

[2] Golebiewska EM, Poole AW. Platelet secretion: From haemostasis to wound healing and beyond. Blood Rev. 2015;29(3):153–62. doi: 10.1016/j.blre.2014.10.003 ; PubMed Central PMCID: PMC4452143. 25468720 10.1016/j.blre.2014.10.003 PMC4452143

[3] Harrison P, Mackie I, Mumford A, Briggs C, Liesner R, Winter M, et al Guidelines for the laboratory investigation of heritable disorders of platelet function. Br J Haematol. 2011;155(1):30–44. doi: 10.1111/j.1365-2141.2011.08793.x . 21790527 10.1111/j.1365-2141.2011.08793.x

[4] Fratantoni JC, Poindexter BJ. Measuring platelet aggregation with microplate reader. A new technical approach to platelet aggregation studies. American journal of clinical pathology. 1990;94(5):613–7. . 2239825 10.1093/ajcp/94.5.613

[5] Bednar B, Condra C, Gould RJ, Connolly TM. Platelet aggregation monitored in a 96 well microplate reader is useful for evaluation of platelet agonists and antagonists. Thromb Res. 1995;77(5):453–63. . 7778060 10.1016/0049-3848(95)93881-y

[6] Hoylaerts MF, Thys C, Stassen JM, Vermylen J. Dose-responses to inducers and inhibitors of platelet aggregation analysed via a micro-method. Blood coagulation & fibrinolysis: an international journal in haemostasis and thrombosis. 1996;7(2):187–90. . 8735815 10.1097/00001721-199603000-00019

[7] Salmon DM. Optimisation of platelet aggregometry utilising micotitreplate technology and integrated software. Thromb Res. 1996;84(3):213–6. . 8914222 10.1016/0049-3848(96)00179-x

[8] Walkowiak B, Kesy A, Michalec L. Microplate reader—a convenient tool in studies of blood coagulation. Thromb Res. 1997;87(1):95–103. . 9253804 10.1016/s0049-3848(97)00108-4

[9] Moran N, Kiernan A, Dunne E, Edwards RJ, Shields DC, Kenny D. Monitoring modulators of platelet aggregation in a microtiter plate assay. Analytical biochemistry. 2006;357(1):77–84. doi: 10.1016/j.ab.2006.06.037 . 16920064 10.1016/j.ab.2006.06.037

[10] Chan MV, Armstrong PC, Papalia F, Kirkby NS, Warner TD. Optical multichannel (optimul) platelet aggregometry in 96-well plates as an additional method of platelet reactivity testing. Platelets. 2011;22(7):485–94. doi: 10.3109/09537104.2011.592958 . 21806492 10.3109/09537104.2011.592958

[11] Chan MV, Warner TD. Standardised optical multichannel (optimul) platelet aggregometry using high-speed shaking and fixed time point readings. Platelets. 2012;23(5):404–8. doi: 10.3109/09537104.2011.603066 . 21806495 10.3109/09537104.2011.603066

[12] Lordkipanidze M, Lowe GC, Kirkby NS, Chan MV, Lundberg MH, Morgan NV, et al Characterization of multiple platelet activation pathways in patients with bleeding as a high-throughput screening option: use of 96-well Optimul assay. Blood. 2014;123(8):e11–22. doi: 10.1182/blood-2013-08-520387 ; PubMed Central PMCID: PMC3931193. 24408324 10.1182/blood-2013-08-520387 PMC3931193

[13] Mustard JF, Perry DW, Ardlie NG, Packham MA. Preparation of suspensions of washed platelets from humans. Br J Haematol. 1972;22(2):193–204. . 4333433 10.1111/j.1365-2141.1972.tb08800.x

[14] Cattaneo M, Cerletti C, Harrison P, Hayward CP, Kenny D, Nugent D, et al Recommendations for the Standardization of Light Transmission Aggregometry: A Consensus of the Working Party from the Platelet Physiology Subcommittee of SSC/ISTH. J Thromb Haemost. 2013;11:1183–93. doi: 10.1111/jth.12231 . 23574625 10.1111/jth.12231

